# Machine learning-based risk prediction model for canine myxomatous mitral valve disease using electronic health record data

**DOI:** 10.3389/fvets.2023.1189157

**Published:** 2023-08-31

**Authors:** Yunji Kim, Jaejin Kim, Sehoon Kim, Hwayoung Youn, Jihye Choi, Kyoungwon Seo

**Affiliations:** ^1^Department of Veterinary Internal Medicine, College of Veterinary Medicine, Seoul, Republic of Korea; ^2^School of Biological Sciences, Seoul National University, Seoul, Republic of Korea; ^3^Department of Veterinary Medical Imaging, College of Veterinary Medicine, Seoul National University, Seoul, Republic of Korea

**Keywords:** canine, artificial intelligence, feature ranking, heart failure, random forest

## Abstract

**Introduction:**

Myxomatous mitral valve disease (MMVD) is the most common cause of heart failure in dogs, and assessing the risk of heart failure in dogs with MMVD is often challenging. Machine learning applied to electronic health records (EHRs) is an effective tool for predicting prognosis in the medical field. This study aimed to develop machine learning-based heart failure risk prediction models for dogs with MMVD using a dataset of EHRs.

**Methods:**

A total of 143 dogs with MMVD between May 2018 and May 2022. Complete medical records were reviewed for all patients. Demographic data, radiographic measurements, echocardiographic values, and laboratory results were obtained from the clinical database. Four machine-learning algorithms (random forest, K-nearest neighbors, naïve Bayes, support vector machine) were used to develop risk prediction models. Model performance was represented by plotting the receiver operating characteristic (ROC) curve and calculating the area under the curve (AUC). The best-performing model was chosen for the feature-ranking process.

**Results:**

The random forest model showed superior performance to the other models (AUC = 0.88), while the performance of the K-nearest neighbors model showed the lowest performance (AUC = 0.69). The top three models showed excellent performance (AUC ≥ 0.8). According to the random forest algorithm’s feature ranking, echocardiographic and radiographic variables had the highest predictive values for heart failure, followed by packed cell volume (PCV) and respiratory rates. Among the electrolyte variables, chloride had the highest predictive value for heart failure.

**Discussion:**

These machine-learning models will enable clinicians to support decision-making in estimating the prognosis of patients with MMVD.

## 1. Introduction

Myxomatous mitral valve disease (MMVD) is the most common cardiovascular disease, accounting for approximately 75% of all canine heart diseases. Moreover, MMVD is the most common cause of heart failure (HF) in small-breed dogs (1–3). The progression of MMVD depends on several risk factors; therefore, the prognosis differs significantly between patients (4). Although there are many preclinical MMVD patients, few develop congestive HF, while others develop various forms of HF or even cardiac death. Furthermore, cardiogenic pulmonary edema due to HF is one of the leading causes of cardiac death in dogs (1).

Given the importance of the disease, predicting HF in patients with MMVD has become a priority. However, this requires a comprehensive interpretation of various clinical data, which is too complex to predict at the right time. Several studies have been conducted to identify key risk factors contributing to the development of HF in dogs with MMVD; however, there are still limitations in predicting the risk of HF (2). Therefore, new methods to support the assessment and prediction of the risk of HF in patients with MMVD are warranted.

In human medicine, many studies have attempted to use advanced technologies to assess and predict the risk and onset of HF and prognosis in cardiovascular diseases (5–8). Electronic health records (EHRs) are considered useful data sources to reveal correlations with clinical data (9). Machine learning, a branch of artificial intelligence applied to medical records, is an effective tool for prognostic prediction and medical decision-making.

In light of advances in machine-learning technologies, machine learning-based models have outperformed conventional risk prediction models owing to their capability to process large volumes and various data types (9, 10). Several recent medical studies have attempted to develop machine learning-based models and have improved the performance of classifiers from simple infectious diseases to complex heterogeneous diseases by using various machine-learning algorithms in human and veterinary medicine (11–15).

This study aimed to develop machine learning-based risk prediction models for HF in dogs with MMVD using a dataset of EHRs. Additionally, four machine-learning algorithms were used, and significant HF predictive markers were identified through feature ranking of the best-performing algorithm.

## 2. Materials and methods

**Figure 1:**
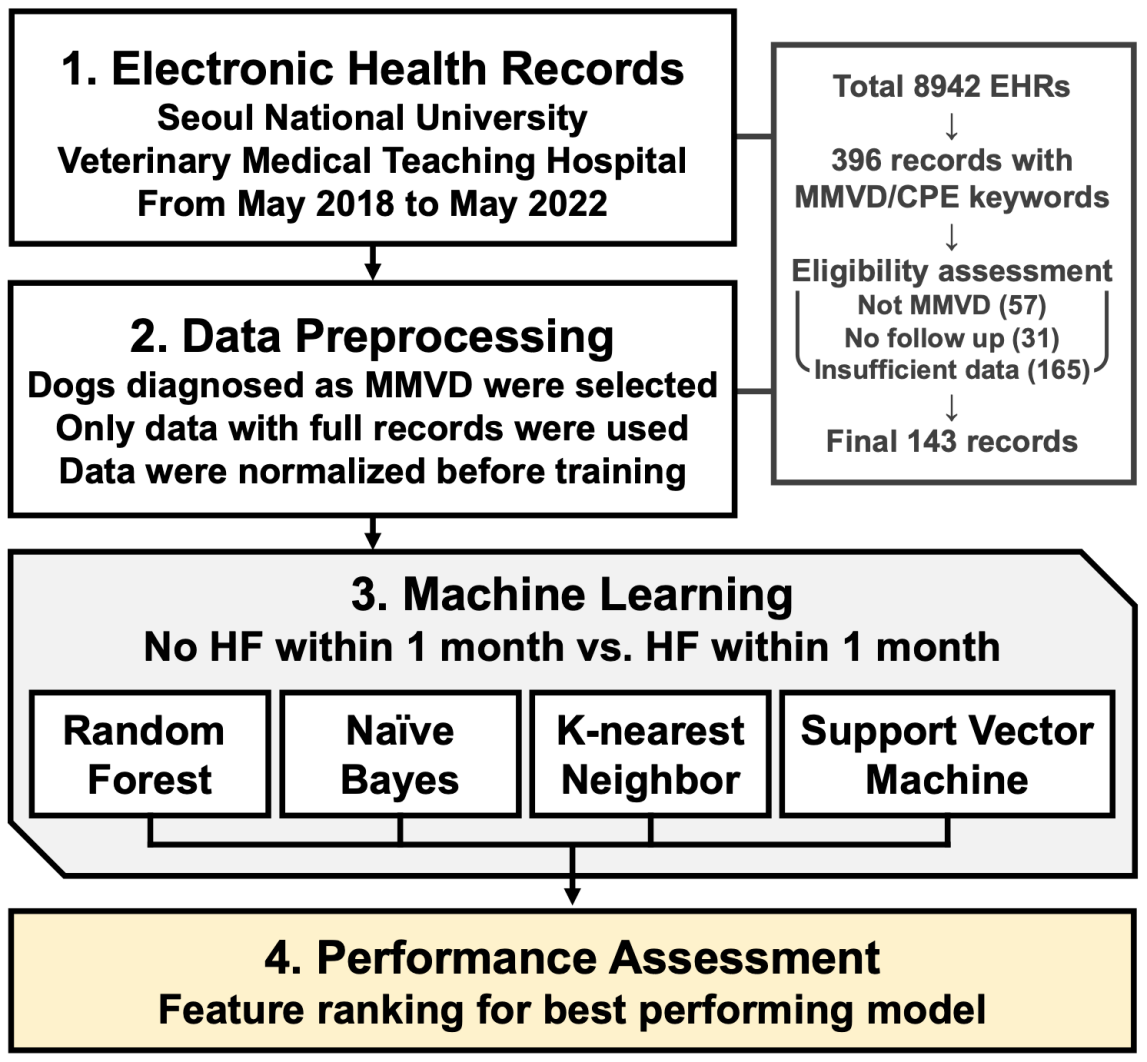
Scheme for risk prediction model and feature ranking.

An illustrative scheme for conducting machine learning-based modeling of HF prediction and feature ranking is shown in Figure 1.

### 2.1. Case selection and data collection

This retrospective study was conducted at the Seoul National University Veterinary Medical Teaching Hospital. The EHRs of 396 records with MMVD were collected between May 2018 and May 2022. All dogs underwent a physical examination, thoracic radiography, echocardiography, and blood analysis. Complete medical records were manually reviewed for all dogs. Demographic data (breed, sex, neuter status, age, and body condition score), radiographic measurements, echocardiographic values, and laboratory results were extracted from the clinical database.

Thoracic radiographic values indicating cardiac remodeling, such as the vertebral heart scale and vertebral left atrial score, were collected. Based on previous echocardiographic measurement studies on the severity and prognosis of MMVD in dogs, five echocardiographic variables were selected for machine learning modeling: the left atrium-to-aorta ratio (LA/Ao ratio), left ventricular end-diastolic diameter normalized for body weight (LVIDDn), left ventricular fractional shortening, E-wave transmitral peak velocity (E-vel), and ejection fraction (2, 16, 17).

As suggested by previous study indicating stable performance with a sample size of around 120, stringent selection criteria was adopted during the data selection process (18). The inclusion criteria were a confirmatory diagnosis of MMVD through radiographic and echocardiographic imaging, with every selected feature available within 1 month of administration or at the time of event. Consequently, out of the 396 initial cases, 165 cases were excluded with insufficient data in either echocardiography or blood test records. Additionally, 31 cases were removed due to the absence of follow-up data, and 57 cases were excluded due to the presence of other cardiac diseases coexisting with MMVD or when the cause of heart failure was not MMVD. This rigorous selection process resulted in 143 high-quality cases being included in the final dataset, ensuring the reliability and robustness of our machine learning analysis.

The patients were divided into MMVD with HF (HF group) and MMVD without HF (non-HF group). Non-HF was defined as when the patients do not have an HF event more than once month. MMVD-related HF was confirmed by cardiogenic pulmonary edema on thoracic radiographs in relation to patient history and clinical assessment. Moreover, patients with MMVD had previous HF episodes and cardiac death.

Cases with complete demographic data, physical examination data, laboratory results, and clinical imaging data were used for further analysis. Features with categorical data were assessed using counts and corresponding percentages, and continuous numerical data were summarized using the mean value.

### 2.2. Machine-learning model development

The data values were first transformed into appropriate data types for machine learning. Continuous variables were normalized using min-max normalization, while binary categorization was applied to neuter status, sex, anemia, and tachypnea. HF events were the target of this binary prediction model. Four frequently used machine-learning algorithms were trained to develop an optimized prediction model: random forest (RF), K-nearest neighbors (KNN), naïve Bayes (NB), and support vector machine (SVM).

RF is an ensemble learning method based on multiple decision trees. The decision trees are randomly built from the variable set. The prediction is made by majority vote across all decision trees (19). KNN is an instance-based model which is based on the characteristic of the K-nearest neighbor of a new point to classify it (20). NB is a probabilistic classifier based on the assumption of independence between the variables of the problem. The NB model performs a probabilistic classification of an unclassified sample to put it in the most likely class (21). SVM is a high-performance model for non-linear problems which discriminates between two classes by generating a hyperplane. SVM is not biased by outliers and is not sensitive to them (22).

The model training process was repeated 100 times to yield an average confusion matrix to tune the hyperparameters. The database was split randomly into a training and testing set for each of the 100 executions to prevent model overfitting. In cases of hyperparameter optimization, the database was split into training, testing, and validation sets wherein the prediction results were measured using confusion matrix rates such as sensitivity, specificity, and accuracy. In addition, model performance was represented by plotting the receiver operating characteristic (ROC) curve and calculating the area under the curve (AUC) (23). The best-performing model was defined by having the highest AUC and was chosen for the feature-ranking process.

### 2.3. Feature ranking

The top-performing model, RF, was used for the feature-ranking process, wherein the Gini impurity-reduction feature-ranking technique was applied (24). Using a dataset, RF constructs multiple random decision trees and checks all binary outcomes across all decision trees. Additionally, RF chooses its final output through a majority vote. Feature ranking is based on the mean Gini decrease value of how much the Gini impurity decreases when a specific variable is removed. The algorithm then compares the Gini value with the other Gini values obtained by applying all other features and ranks the features according to their significance.

### 2.4. Statistical analysis

Statistical analyzes and machine learning were conducted using R version 4.2.0 (R software, R Core Team, Vienna, Austria) and various R packages (class, clusterSim, dplyr, e1071, formula.tools, gmodels, kernlab, pastecs, PRROC, randomForest, ROCR, ROSE, rpart), while Prism 9 (GraphPad Software, San Diego, CA) was used to create graphs. All codes and data used for this analysis are provided upon request.

## 3. Results

### 3.1. Patient characteristics

Among 143 patients, 90 (63%) were labeled as MMVD with HF and 53 (37%) as MMVD without HF. According to the American College of Veterinary Internal Medicine classification, the HF and non-HF groups were classified as stages B and C/D, respectively.

Although 25 variables were collected from the EHRs, only 24 were included in the dataset since the breed variable was removed due to dataset encoding complexity. Of the dogs in the HF group, 48 (53.33%) were male and 42 (46.67%) were female, with a mean age of 11.78 years. Of the patients in the non-HF group, 30 (56.6%) were male and 23 (43.4%) were female, with a mean age of 11.45 years. Several breeds of dogs were included in this study. For the HF group, 15 breeds were recorded, with Maltese being the most frequently observed (50%), followed by Shih Tzu (11%) and Pomeranian (10%); 26 dogs were of 12 other breeds. Thirteen different breeds were included in the non-HF group, with Maltese (24%) being the most observed, followed by Poodle (7%), Shih Tzu (4%), and Chihuahua (4%). There were fewer anemia and tachypnea cases in the non-HF group, while there were more HF cases. The demographic and binary variables for each group are summarized in Table 1.

**Table 1:** Summary of patient demographics and binary features of dogs in the dataset.

Category feature	Heart failure (*n* = 90)		Non-heart failure (*n* = 53)	
	#	%	#	%
Sex				
Male	48	53.33	30	56.6
Female	42	46.67	23	43.4
Neuter status				
Entire	12	13.33	2	3.77
Neutered	78	86.67	51	96.23
Anemia				
Anemia	22	24.44	1	1.89
Normal	68	75.56	52	98.11
Tachypnea				
Tachypnea	65	72.22	20	62.26
Normal	25	27.78	33	37.74
Breed				
Maltese	45	50	24	45.28
Shih-Tzu	10	11.11	4	7.54
Poodle	3	3.33	7	13.2
Pomeranian	9	10	2	3.77
Chihuahua	3	3.33	4	7.54
Yorkshire Terrier	2	2.22	2	3.77
Pekinese	3	3.33	1	1.88
Cocker Spaniel	2	2.22	0	0
Spitz	2	2.22	2	3.77
Bichon Frise	1	1.11	1	1.88
Schnauzer	1	1.11	1	1.88
Mixed	6	6.66	2	3.77
Others	3	3.33	3	5.66

The mean thoracic radiographic and three echocardiographic values (LA/Ao ratio, LVIDDn, and E-vel) were higher in the HF group. In addition, the mean of each electrolyte feature was similar between groups, except for chloride. The quantitative characteristics of the datasets are presented in Table 2.

**Table 2:** Statistical quantitative description of the numeric features of dogs in the dataset.

Numeric Feature	Heart failure (*n* = 90)			Non-heart failure (*n* = 53)				Statistics	
	Median	Mean	Range	Median	Mean	Range	*p*-value (JB)	*p*-value (ST)	*p*-value (MW)
Age	12	11.78	[3–18]	11	11.45	[6–19]	0.77	0.49	–
VHS	11.4	11.44	[9.2–14.7]	10.5	10.47	[9–12.4]	0.00	–	0.00
VLAS	2.8	2.79	[1.5–4.1]	2.4	2.39	[1.6–3.2]	0.18	0.00	–
Cl (mmol/L)	112	111.62	[91–127]	115.1	114.67	[104.1–123.5]	0.00	–	0.00
K (mmol/L)	4.31	4.33	[2.62–6.37]	4.5	4.57	[2.87–5.87]	0.11	0.04	–
Na (mmol/L)	144.7	145.54	[125–178]	145.6	145.53	[135.6–156]	0.00	–	0.25
LVIDDn	1.94	1.92	[0.73–2.64]	1.6	1.59	[1.11–2]	0.45	0.00	–
LA/Ao ratio	1.83	1.87	[1.1–3.58]	1.5	1.55	[1.18–2.4]	0.00	–	0.00
E-vel (m/s)	1.34	1.32	[0.39–2.72]	0.94	0.98	[0.46–1.74]	0.01	–	0.00
EF (%)	90.75	89.3	[64.5–99.4]	91.7	91.09	[74.9–98.5]	0.00	–	0.23
FS (%)	57.35	57.47	[33.9–81.4]	56.8	59.18	[42.6–88]	0.65	0.32	–
PCV (%)	41.5	41.21	[19.3–57.2]	45.7	45.53	[32.1–58.4]	0.41	0.00	–
BUN (mg/dL)	28.9	34,1	[3.86–115.5]	22	23.93	[1.43–58.4]	0.00	–	0.00
Serum Cr (mg/dL)	1.13	1.26	[0.5–5.57]	0.92	1.01	[0.33–2.08]	0.00	–	0.01
Systolic BP (mmHg)	120	125.02	[50–200]	135	136.3	[100–180]	0.63	0.01	–
Murmur	4	4.3	[3–6]	4	3.98	[0–6]	0.00	–	0.01
BCS	5	4.61	[2–8]	5	5.13	[2–8]	0.22	0.01	–
Respiratory rate	45	54.56	[18–120]	30	36.83	[12–100]	0.00	–	0.00
Platelet (10,000/μL)	45.9	49.81	[4.7–175.9]	43	45.91	[13.6–71.9]	0.00	–	0.38
Temperature (°C)	38.6	38.54	[36.8–40.3]	38.5	38.53	[37.1–39.6]	0.62	0.93	–

BCS, body condition score, BUN, blood urea nitrogen; PCV, packed cell volume; EF, ejection fraction; FS, fractional shortening; LA/Ao ratio, left atrium to aorta ratio; LVIDDn, left ventricular end-diastolic diameter normalized for body weight; E-vel, E-wave transmitral peak velocity; VHS, vertebral heart scale; VLAS, vertebral left atrial score; JB, Jarque-Bera test; ST, Student’s t-test; MW, Mann-Withney test.

### 3.2. Machine-learning performance evaluation

**Figure 2:**
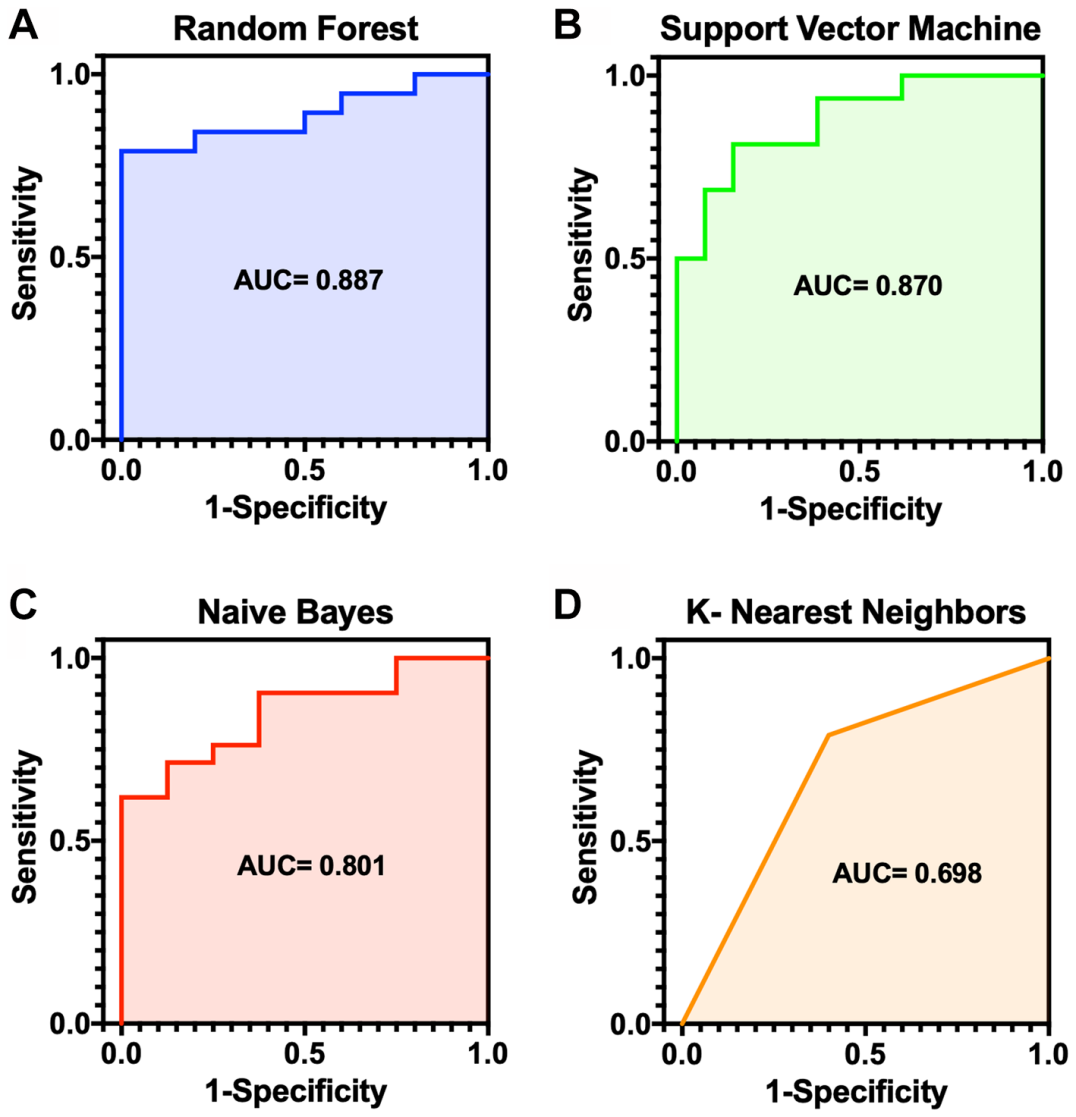
ROC curves for the risk prediction models for MMVD dogs. **(A)** Random forest (AUC 0.887). **(B)** Support vector machine (AUC 0.870). **(C)** Naïve bayes (AUC 0.801). **(D)** K-nearest neighbors (AUC 0.698). AUC, area under the curve; MMVD, myxomatous mitral valve disease; ROC, receiver operator characteristic.

For algorithms that required hyperparameter optimization, such as KNN and SVM, the dataset was randomly split into 60, 20, and 20% for the training, validation, and test sets, respectively. On the contrary, the other algorithms, RF and NB, split the dataset into 80% for the training set and 20% for the test set. The model prediction results were reported as accurate, sensitive, and specific. ROC plots were generated and AUCs were calculated to estimate model discrimination. The mean result scores of the four methods are demonstrated in Table 3. Figure 2 displays the ROC curves for the four machine-learning models. Of the four methods, RF showed superior performance to the other models in terms of accuracy (0.78), sensitivity (0.85), and AUC (0.88). NB showed the highest specificity (0.87) compared to RF (0.68). The top three models indicated very good performance (AUC ≥ 0.8) in the dataset, while the performance of KNN showed the lowest AUC (0.69).

**Table 3:** Performance of ML models predicting heart failure risk of MMVD dogs – mean of 100 executions.

Methods	Accuracy	Sensitivity	Specificity	AUC
Random Forest	0.784	0.855	0.689	0.887
Naïve Bayes	0.781	0.724	0.878	0.801
Support Vector Machine	0.764	0.82	0.69	0.87
K-nearest Neighbors	0.718	0.799	0.598	0.698

ML, machine learning; MMVD, myxomatous mitral valve disease.

### 3.3. Feature selection results

**Figure 3:**
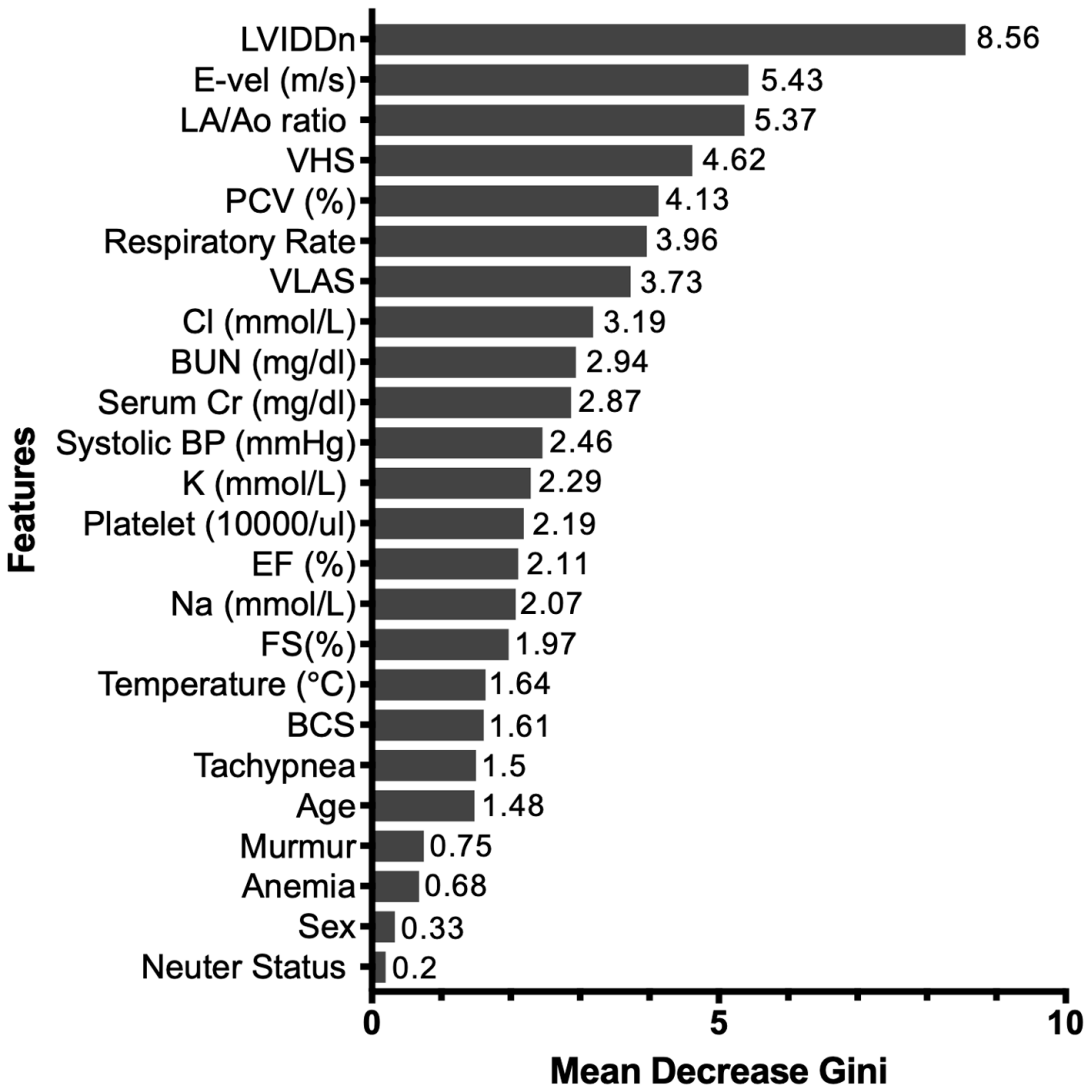
Random-forest feature-ranking selection through mean Gini decrease value.

The RF algorithm, which was the top-performing model, was used for feature-ranking analysis. The mean Gini decrease value was used to rank the significance of its variables and was listed in the order of importance. The most influential predictor was LVIDDn, followed by LA/Ao ratio and E-vel. Furthermore, both thoracic radiographic values were highly predictive of HF. Among the electrolyte features, chloride had the highest predictive value for HF. Respiratory rate and packed cell volume (PCV) were selected based on their relative influence compared to anemia and tachypnea. Neuter status and sex were last in the feature ranking. Figure 3 shows the feature ranking selected by RF.

## 4. Discussion

In this study, four supervised machine-learning models were developed to predict the risk of HF in dogs with MMVD using EHRs. Several machine-learning algorithms can be used to analyze diseases, each with advantages and disadvantages. This study considered algorithms most frequently used to achieve the best performance. Other studies also showed that RF had superior performance compared to other methods (25–27). The feature-ranking method used RF because of its high evaluation results and efficiency for identifying novel risk predictors and complex interplay between variables (28–30).

The input data quality can strongly influence the performance of a machine-learning model (10). Overall, all classifiers performed well in this study. The RF model outperformed all other methods with the highest AUC; the KNN model demonstrated a decline in performance but was still acceptable. This finding indicates that the dataset is ideal for developing a risk assessment model for canine MMVD, thus suggesting that machine learning is effective in assessing the preclinical risk of MMVD patients developing HF and death due to underlying cardiac disease.

The degree of disease progression often differs between patients because of heterogeneous features and MMVD manifestations. Moreover, the high burden of comorbidities and unpredictable, complex interactions makes it challenging to assess and establish treatment strategies. Improved identification of patients with HF could provide opportunities to detect patients early on and provide appropriate monitoring strategies. Therefore, the classification of whether there is a risk of HF itself can help significantly in the management of patients.

According to the demographic characteristics, the disease pattern is similar to that of other studies, demonstrating a higher percentage of male and smaller (<20 kg) breeds (3). In this study, the number of male dogs was slightly higher than that of female dogs in both groups. Age is also considered a contributing factor to disease development in dogs. Several studies have shown a high prevalence of MMVD associated with aging (2, 4). Similarly, in this study, both groups had a mean age of approximately 11 years, indicating they belonged to a senior population. However, while the data showed a certain extent of older age, other factors are considered to have more predictive power than age in this model. Echocardiographic and radiographic variables had the highest predictive value for HF. The features with the most significant influence on the model were LVIDDn, LA/Ao ratio, and E-vel, which are echocardiographic features that have been proposed as contributing factors to the severity of MMVD (16, 31). The prognostic value of these echocardiographic features was higher than that of thoracic radiographic measurements, which means that the algorithm provided more value when assessing HF risk.

Numeric features (PCV and respiratory rates) showed a higher mean Gini decrease value than binary features (anemia and tachypnea). The criteria for a binary diagnosis of anemia or tachypnea focus on normal patients. Therefore, the risk level can generally be judged only by existing criteria. However, there may be considerable risk in the case of a patient who is more sensitive to a specific stress, even if it falls within the normal range according to existing criteria. Therefore, it is necessary to reconsider the diagnostic criteria for anemia and tachypnea in patients with MMVD. Considering the multifactorial nature of the disease, this implies that careful assessment of the numeric value is required when the clinical examination results are on the borderline of the normal reference range rather than whether the patients have anemia and tachypnea.

Among the electrolyte variables, chloride showed the highest mean Gini decrease value. Electrolyte abnormalities, including hyponatremia and hypochloremia, can be observed in patients with congestive HF. A recent retrospective study reported that hypochloremia was considered a negative prognostic marker in dogs with HF (32). Concurrent renal impairment and HF, defined as a cardiorenal syndrome, has a negative prognostic impact on patients (33, 34). Similarly, serum creatinine has a moderate predictive value in this study. Since dog sizes vary depending on breed and weight, this study used body condition scores to reflect the overall body condition of individual dogs; however, these scores had a relatively lower predictive value in the feature-ranking results.

This study has some limitations. First, due to the imbalance of the dataset, most models obtained better performance in terms of sensitivity than specificity. These results occurred since algorithms were exposed to more HF patient components than those of non-HF patients during training; hence, they are more equipped to recognize HF patient profiles during testing. Second, treatment was not considered, which may have resulted in underestimating certain clinical values, thereby influencing the results. Further investigation on additional external datasets with the same variables from a different cohort of dogs would improve prediction performance.

The most common cardiovascular disease among small-breed dogs is MMVD, and small-breed dogs tend to be prone to developing MMVD (1). Given that most breeds presented in this dataset are small-breed patients (weight ≤ 15 kg), our results are consistent with the prevalence of MMVD documented in previous studies (1, 4). Therefore, the model outputs are optimized for small-breed dogs rather than large-breed dogs. Moreover, the prevalence of heart disease is different in large-breed dogs (e.g., canine dilated cardiomyopathy). Further access to different heart diseases in large-breed patients would enable the development of other risk stratification models.

Patient EHRs are stored with each admission, and diagnostic variables may change over time. Since time-varying values have more evidence for the determination of HF, advanced machine-learning techniques such as recurrent neural networks may show better predictive power by reflecting all the periodic chart data (35). However, training a recurrent neural network with a complex dataset is very difficult and requires significant computing power for each patient, making it more difficult for use in real clinical practice. Even though our RF model was trained with only baseline data regardless of previous history records, it shows very good performance (AUC = 0.88). Our model requires much less computing power and is relatively easy to train for predicting patients at high risk of HF. Therefore, this platform can be further applied to predict significant HF events during disease management.

The data used in this study were extracted from referral animal hospitals, which means that patients may have more complicated clinicopathologic characteristics and different tendencies than general MMVD patients; therefore, known prognostic factors may not be well applied. However, machine-learning algorithms consider correlations between large volumes of variables, and this process contributes to the increased ranking of other undervalued features in general MMVD patients. This can assist the drive toward personalized risk management and provide insight into the veterinarian’s decision-making for patients with MMVD.

In summary, this study obtained significant results in predicting HF events in patients with MMVD. Although further testing and validation are needed for incorporation into clinical practice, this study highlights the potential of machine learning in heart disease management and can encourage future approaches to apply machine learning in veterinary medicine. Simultaneously, a more comprehensive application of machine learning may improve diagnosis and treatment decisions for diseases and risk prediction.

## Data availability statement

The raw data supporting the conclusions of this article will be made available by the authors, without undue reservation.

## Author contributions

YK, JK, and KS contributed to study design, data analysis, and interpretation. YK and JK wrote and edited codes for machine learning. SK contributed to data interpretation, manuscript review, and editing. KS, HY, and JC contributed to manuscript review and editing. YK worte the original draft. All authors contributed to the article and approved the submitted version.

## Conflict of interest

The authors declare that the research was conducted in the absence of any commercial or financial relationships that could be construed as a potential conflict of interest.

## Publisher’s note

All claims expressed in this article are solely those of the authors and do not necessarily represent those of their affiliated organizations, or those of the publisher, the editors and the reviewers. Any product that may be evaluated in this article, or claim that may be made by its manufacturer, is not guaranteed or endorsed by the publisher.
